# Traumatic deep vein thrombosis in a soccer player: A case study

**DOI:** 10.1186/1477-9560-2-8

**Published:** 2004-10-14

**Authors:** Paul S Echlin, Ross EG Upshur, Douglas B McKeag, Harsha P Jayatilake

**Affiliations:** 1 Providence Athletic Medicine, Providence Hospital and Medical Centers, 47601 Grand River Avenue, Suite 101, Novi Michigan, United States of America 48374; 2 Primary Care Research Unit, Department of Family and Community Medicine, Sunnybrook and Women's College Health Sciences Centre, 2075 Bayview Avenue, #E-349, Toronto, Ontario, Canada M4N 3M5; 3 Department of Family and Community Medicine, University of Toronto, 263 McCaul Street, Toronto, Ontario, Canada M5T 1W7; 4 Department of Public Health Sciences, University of Toronto, 12 Queen's Park Crescent W., Toronto, Ontario, Canada M5S 1A8; 5 Department of Family Medicine, Indiana University, Long Hospital, Second Floor. 1110 West Michigan Street, Indianapolis Indiana, United States of America 46202-5102; 6 Department of Family Medicine, Wayne State University, 15400 West McNichols, 2^nd ^Floor, Detroit, Michigan, United States of America 48235

## Abstract

A 42 year-old male former semi-professional soccer player sustained a right lower extremity popliteal contusion during a soccer game. He was clinically diagnosed with a possible traumatic deep vein thrombosis (DVT), and sent for confirmatory tests. A duplex doppler ultrasound was positive for DVT, and the patient was admitted to hospital for anticoagulation (unfractionated heparin, warfarin). Upon discharge from hospital the patient continued oral warfarin anticoagulation (six months), and the use of compression stockings (nine months). He followed up with his family doctor at regular intervals for serial coagulation measurements, and ultrasound examinations. The patient's only identified major thrombotic risk factor was the traumatic injury. One year after the initial deep vein thrombosis (DVT) the patient returned to contact sport, however he continued to have intermittent symptoms of right lower leg pain and right knee effusion.

Athletes can develop vascular injuries in a variety of contact and non-contact sports. Trauma is one of the most common causes of lower extremity deep vein thrombosis (DVT), however athletic injuries involving lower extremity traumatic DVT are seldom reported. This diagnosis and the associated risk factors must be considered during the initial physical examination. The primary method of radiological diagnosis of lower extremity DVT is a complete bilateral duplex sonography, which can be augmented by other methods such as evidence-based risk factor analysis. Antithrombotic medication is the current standard of treatment for DVT.    Acute thrombolytic treatment has demonstrated an improved therapeutic efficacy, and a decrease in post-DVT symptoms.

There is a lack of scientific literature concerning the return to sport protocol following a DVT event. Athletic individuals who desire to return to sport after a DVT need to be fully informed about their treatment and risk of reoccurrence, so that appropriate decisions can be made.

## Introduction

Athletes are susceptible to a variety of vascular injuries, secondary to either repetitive motion, or high-speed collisions [1]. The differential diagnosis for lower extremity trauma in sport seldom invites a diagnosis of vascular injury, such as a deep vein thrombosis (DVT). Failure of the physician to recognize a vascular injury can have catastrophic limb or life threatening (pulmonary embolism) implications. The epidemiology, diagnosis, treatment, and recurrence of DVT, as well as the prevention of post-thrombotic symptoms are the most current areas of clinical research. Research-based guidelines concerning an athlete's return to sport after a DVT is an important area for future investigation.

## Case Report

A 42 year old Polish born male former semi-professional soccer player was seen on May 16^th^, 2003 in the emergency department, with the chief complaint of right leg pain. The patient had been playing soccer 10 days prior to this visit, and recalled a traumatic "tackle" injury to the posterior area of his right lower extremity. He denied experiencing any sensation of tearing or popping in the right knee during the index trauma, and was able to complete the game with only minor discomfort. On day 3 post-injury the patient noted severe pain in his knee and calf with ambulation. The patient visited his primary doctor on post-injury day 8 and was diagnosed with a right lower extremity soft tissue injury. A right lower extremity echo-doppler ultrasound (US), and a semi-quantitative D-dimer automated latex procedure were ordered to rule out a vascular disorder. The US investigation demonstrated a DVT in the distal femoral, popliteal, and distal calf veins, with a heterogenous mass (5 cm × 3 cm × 4 cm, resembling a hematoma) without a doppler signal in the right popliteal fossa. The D-dimer result was also positive for a suspected thrombosis (1.0–2.0 ug/ml; range = <0.25 ug/ml). The patient was instructed by his physician to proceed immediately to the emergency department for further evaluation and treatment.

The past medical and family history of the patient was non-contributory for a history of thrombophilia or other thrombotic major risk factors. The patient had a remote (11 years old) surgical history of a right-sided inguinal hernia that could have created scar tissue contributing to vascular obstruction and stasis. The initial emergency department examination demonstrated an exquisitely tender right calf with a 3 cm difference in mid-calf girth (10 cm. distal from each inferior patellar pole); a 1+ right knee supra patellar effusion; and a palpable popliteal mass with visible ecchymosis. Laboratory tests (CBC, Lytes, PT, PTT, ESR, CPK, Anti-throbomin, Factor V Leiden, Lupus Screen, ANA, Anti-Cardiolipin, Protein C, and Protein S) were negative for metabolic, hematological or familial abnormalities. A repeat US investigation confirmed the results of the previous outpatient results. The patient was anticoagulated simultaneously with unfractionated heparin and Warfarin sulfate. A multiview plain film x-ray examination of the right lower extremity demonstrated no fracture, dislocation, or bony mass.

A magnetic resonance image (MRI) of the right knee was done several days after admission, to verify a torn right knee meniscal cartilage that had been previously diagnosed. The official MRI radiological report included a small free-edge tear of the posterior horn root junction of the lateral meniscus, chondromalasia (lateral patella and lateral femoral articular cartilage), and a moderate joint effusion with a bursal cyst or dilated semimembranous-gastronemius bursa.

Anticoagulation was achieved on day 6 of the patient's hospitalization. He was discharged on 5 mg of warfarin per day, with instructions to continue the use of compression stockings. The patient was also advised to follow up with his primary physician for regular monitoring, and to avoid contact or collision activities during anticoagulation.

The patient was maintained on warfarin for six months, with weekly physician monitoring (symptoms, PT, INR) for the first three months post-injury. The monitoring interval was changed to once per month for the remainder of the treatment period. Hematologic investigations (APTT, PT, INR, Cardiolipin antibody, C-reactive protein, Lupant anticoagulant, Factor V Leiden, Antithrombin, ANA, Protein C, Protein S, and RPR) were obtained three months post injury. There were no contributory thrombophilic factors found in these investigations. Laboratory levels of Protein C activity 22% (range = 70–140%), Protein S activity 48% (range = 75–140%), INR 2.57 (range = 0.88–1.12), and PT 27.5 sec (range = 9.6–12.0 sec); APTT 38.5 sec (range = 23.4–35.4 sec) were found to be appropriately reactive to the anticoagulant therapy.

The patient underwent two arthrocentesis procedures to remove small amounts of serous fluid from the joint, and each time was injected with a lidocaine/corticosteroid combination. US examinations after the hospitalization period failed to demonstrate a recurrence or new onset of DVT, however residual echogenic material characteristic of a chronic thrombus was demonstrated in the popliteal vein. Compression stocking use was maintained after hospital discharge, and was discontinued after nine months. The patient returned to soccer after anticoagulation, with a full understanding of his increased risk of DVT recurrence.

One-year post injury the patient continued to suffer from intermittent right lower extremity discomfort and swelling often unrelated to activity. An elective arthroscopy was recently performed on the patient's right knee to investigate his long-standing meniscal disruption and effusion. The arthroscopy demonstrated several areas of arthrosis (patellar lateral and medial facets, lateral and medial femoral condyles), and a torn lateral meniscus. Appropriate partial lateral menisectomy and debridement, and chondroplasty of the areas of arthrosis were preformed. An arthroscopic examination of the posterior compartment demonstrated a small cleft-like area just medial to the semimembranosis where the Baker's cyst likely originated. The patient returned to the orthopedist one week post-op with a large (150 cc's) hemarthrosis that was aspirated from the knee. He was requested to follow-up in one month for re-evaluation.

## Discussion

This case study illustrates the importance of considering deep vein thrombosis in the diagnosis of sport-related extremity trauma. DVT is classically related to venous stasis, intimal injury, and coagulation diathesis (Virchow's triad). The estimated incidence of DVT from all causes is 0.5 to 1.6 per 1000 persons per year, and may be an underestimation due to the number of DVT that are asymptomatic [2].

Standard risk factors for DVT are immobilization, pregnancy, recent surgery (particularly orthopedic), malignancy, older age, smoking, coagulation deficits or hypercoagulable states, connective tissue disorders, sex steroid administration, severe dehydration, and major trauma. Bates et al. [3] presented a table of the estimated relative risk (RR) for individual DVT risk factors. These factors include inherited conditions (e.g. Factor V Leiden, RR = 50, Antithrombin deficiency, RR = 25, Protein C and S deficiency, RR = 10); acquired conditions (e.g. major surgery or trauma, RR = 5–200; history of venous thromboembolism, RR = 50); and hereditary, environmental, or idiopathic conditions (e.g. hyperhomocysteinemia, and elevated levels of Factor VIII, RR = 3: elevated levels of Factor IX, RR = 2.3).

Coagulation diathesis through congenital or acquired thrombophilia may promote coagulation [3]. Coagulation deficits in previously healthy athletes are becoming increasingly identified through laboratory tests, and must be considered as contributing factors for DVTs [4-7]. Hilberg et al. [6] found that the risk of hereditary exists in elite athletes, corresponds to the general population. These authors proposed that countermeasures (e.g. early anticoagulation during periods of immobilization/injury; single dose of low molecular weight heparin and/or leg exercises on long-distance flights; and avoiding hemoconcentration with adequate hydration) for athletes who are carriers of a congenital coagulation deficit [6].

The testing for hypercoagulable states in an individual after a single episode of thrombosis is a costly, yet routine procedure in many centers. The common assumption that an identified presence of a thrombophilic abnormality increases the risk of recurrence, and justifies prolonged therapy is without clear supportive evidence. A review of the current literature concerning the treatment of individuals with coagulation deficits concludes that there is no clear evidence that modifying treatment because of an identified hypercoaguable state alters the outcome, or that more intensive therapy is required in patients with laboratory evidence of thrombophillia [3].

Exercise is thought to act as a protective mechanism against thrombosis, due to the controlled balance between the exercise activated coagulation and fibrinolytic pathways [8]. Upper extremity thrombosis that is not related to primary diseases or well known risk factors are rare (2–4% of DVTs).This type of thrombosis has been documented in a variety of sports as effort thrombosis or "Paget-Schroetter's syndrome" [9-14]. This syndrome is been described as a primary thrombosis of the subclavicular and axillary veins, usually proceeded by a strenuous effort or repetitive action involving retroversion and hyperabuction of the extremity [10]. Vascular compression by adjoining bone, ligament and muscle or resulting intimal traumas have been documented as contributing factors toward the development of upper and lower extremity thrombosis [15-27].

Lower extremity DVT with a traumatic sporting injury in otherwise healthy active adults is seldom mentioned in the medical literature [16-29]. This lack of reported cases of this type of thrombosis may be due to either underreporting or incorrect diagnosis. Very few cases of sport-related lower extremity DVT involved direct externally trauma [29,30]. There is one case report (Finnish language) that specifically related DVT development to soccer-related trauma [30], and one case report of lower extremity DVT in a soccer player with coagulation deficiencies [31]. There is also one case report in the literature of a traumatic popliteal thrombosis in a hockey player, which resulted in a fatal pulmonary embolism (PE) [29].

The popliteal, posterior tibial and peroneal veins are susceptible to intimal trauma by the sudden hyperextension and torsion that the lower extremity experiences in a soccer "kick" or "tackle" motion. The popliteal arteries and veins are susceptible to direct, sheering, and muscular compressive forces due to their anatomical position, especially with rapid knee hyperextension or anterior dislocation [13,22].

The literature demonstrates the importance and efficacy of a complete bilateral duplex sonography as the primary method of DVT diagnostic investigation [32]. US findings can be augmented by other methods (e.g. evidence-based risk factor analysis) [33,34]. A review of the current literature also suggests the need for comprehensive evidence-based guidelines concerning the use of radiological diagnostic investigations of suspected DVT [35].

Anticoagulation is effective in preventing DVT propagation and PE, but has no chemical fibrinolytic activity. This type of therapy allows for intrinsic fibrinolysis to occur. Radiographically demonstrable clot lysis occurs in only 50% of anticoagulated patients, and the incidence of complete resolution is less than 5%. Intrinsic fibrinolysis that occurs slowly does not preserve the function of the venous valves, which become fibrotic and fixed after a few weeks of being trapped in clot [36].

The symptoms experienced by individuals without complete clot resolution include heavy or achy legs, edema, throbbing paresthesia, purities, numbness, stiffness, and difficulty standing or ambulating. Postthrombotic syndrome (PTS) is characterized by brawny edema of the leg, stasis dermatitis, hyperpigmentation, induration, ulceration and chronic leg pain. This syndrome is associated with an extraordinary level of chronic pain and disability, and approximately 40% of the total cost of treating DVT is spent on PTS [36].

Zeigler et al. [37] investigated the long-term clinical outcome of individuals with a first DVT. These authors found that 82% of the patients suffered from recurrent symptoms, with a mean follow-up period of 6.6 years. Four level DVT, calf vein thrombosis, recurrence of ipsilateral DVT, and a non-sufficient oral anticoagulation are of prognostic significance for developing clinically relevant symptoms within 10 to 20 years after the first DVT [37].

There is growing evidence that the early lysis provided by thrombolytic therapy is more likely to preserve valve function, decreasing the likelihood of DVT recurrence, and the occurrence of PTS [38,39]. Recent trials of new antithrombotic agent used an endpoint of 'symptomatic recurrent DVT', which was defined as the combination of persistent or recurrent symptoms along with the radiographic evidence of primary clot progression or new thrombus formation. The rate of symptomatic recurrent DVT was reported to be between 4–7%, and does not reflect those individuals who simply continue to be symptomatic after the primary event [35].

The general knowledge concerning quality of life and burden of illness in patients with persistent post-DVT symptoms is limited. This issue is especially important to the athletic patient, as participation in sport is usually an extremely important component of quality of life. For routine monitoring of outcomes in chronic venous disorders there are questionnaires that are available [40,41]. Hedner et al. [42] have recently developed an instrument that measures health and treatment-related quality of life factors in DVD patients.

The athlete's primary concern upon the initial DVT diagnosis is return to play. The issue of return to sport after a lower extremity DVTs has only been addressed only once in the literature concerning return to non-contact sport [43]. General guidelines for sedentary individuals allow for a gradual return to return to daily activities over a six week period [43], with no contact activities allowed during the period of anticoagulation. Roberts and Christie [43] provided a theoretical framework, based on the natural history of animal models for the safe and expeditious return of the athlete. These authors suggested a protocol that combines a graduated return to activity and anticoagulation therapy with regular physician based reevaluation [43].

An athlete who wants to return to a contact or collision sport should be informed of the possible increased risk of recurrent DVT that he or she may face, above the current estimates derived from the general population. There is no current evidence in the literature that investigates the specific risk factor of a traumatic collision, and the recurrence of a DVT. This lack of evidence suggests that the patient and physician should work together to make an informed return to play decision involving the patient's current individual risk profile, the likelihood of DVT recurrence, athletic goals, and the perceived importance of the particular sport to quality of life.

The potential limitations of this case study include the lack of testing for prothrobin mutation, and fibrynolitic parameters (level of tPA, PAI-1 or PAI-1 polymorphism 4G/5G).

## Competing Interests

The author(s) declare that they have no competing interests.

## Author Contributions

PE developed, researched, wrote and revised the case study; RU assisted in study development and manuscript revision; DM assisted in manuscript development and revision; HJ assisted in manuscript development and revision.
